# Identification of recombinant Fabs for structural and functional characterization of HIV-host factor complexes

**DOI:** 10.1371/journal.pone.0250318

**Published:** 2021-05-13

**Authors:** Natalia Sevillano, Evan M. Green, Jörg Votteler, Dong Young Kim, Xuefeng Ren, Bei Yang, Xi Liu, André Luiz Lourenço, James H. Hurley, Shauna Farr-Jones, John D. Gross, Yifan Cheng, Charles S. Craik

**Affiliations:** 1 Department of Pharmaceutical Chemistry, University of California, San Francisco, California, United States of America; 2 Department of Biochemistry and Biophysics, University of California, San Francisco, California, United States of America; 3 Department of Biochemistry, University of Utah, Salt Lake City, Utah, United States of America; 4 Department of Molecular and Cellular Biology, University of California, Berkeley, California, United States of America; 5 Department of Anesthesia and Perioperative Care, University of California, San Francisco, California, United States of America; 6 Howard Hughes Medical Institute, University of California, San Francisco, California, United States of America; New York State Department of Health, UNITED STATES

## Abstract

Viral infection and pathogenesis is mediated by host protein—viral protein complexes that are important targets for therapeutic intervention as they are potentially less prone to development of drug resistance. We have identified human, recombinant antibodies (Fabs) from a phage display library that bind to three HIV-host complexes. We used these Fabs to 1) stabilize the complexes for structural studies; and 2) facilitate characterization of the function of these complexes. Specifically, we generated recombinant Fabs to Vif-CBF-β-ELOB-ELOC (VCBC); ESCRT-I complex and AP2-complex. For each complex we measured binding affinities with K_D_ values of Fabs ranging from 12–419 nM and performed negative stain electron microscopy (nsEM) to obtain low-resolution structures of the HIV-Fab complexes. Select Fabs were converted to scFvs to allow them to fold intracellularly and perturb HIV-host protein complex assembly without affecting other pathways. To identify these recombinant Fabs, we developed a rapid screening pipeline that uses quantitative ELISAs and nsEM to establish whether the Fabs have overlapping or independent epitopes. This pipeline approach is generally applicable to other particularly challenging antigens that are refractory to immunization strategies for antibody generation including multi-protein complexes providing specific, reproducible, and renewable antibody reagents for research and clinical applications. The curated antibodies described here are available to the scientific community for further structural and functional studies on these critical HIV host-factor proteins.

## Introduction

HIV, like other viruses, is an obligate intracellular parasite and requires host cellular machinery at all stages of its life cycle to replicate and counteract the effect of the host immune system. During infection, viruses manipulate the cellular mechanisms of host organisms via pathogen-host interactions to take advantage of the capabilities of host cells, leading to viral replication. Understanding the structure and function of human host factors and host-virus interactions is essential for a complete knowledge of the viral infection and the identification of new targets for therapeutic intervention. However, structural studies of HIV-host complexes have been hindered due to the high degree of flexibility of the complexes, their dynamic nature and the resulting reversibility of complex formation. To assist functional studies, X-ray and cryogenic electron microscopy (EM) structural studies of difficult to study proteins and protein complexes we have developed conformation-specific recombinant antibodies for protein and complex stabilization, capture, and labelling [1–4].

Fabs (fragments antigen binding) have been used to facilitate crystal packing of soluble and integral membrane proteins by binding specific epitopes resulting in stabilization of desired conformations [5–7]. Fabs have also been used in negative stain EM to label domains within a complex [8, 9] and to increase the size of the target protein and provide a fiducial mark with recognizable features for image alignment and better visualization in cryo-EM [3]. Highly specific monoclonal antibodies are usually generated through hybridoma technology or by biopanning with antibody libraries with the target of interest. Non-antigenic sequences in a protein or the need for three-dimensional epitopes can make antigens “hybridoma-refractory” and particularly challenging targets for monoclonal antibody identification since the complexes are unstable and do not maintain their three-dimensional structure during the immunization process.

**Fig 1:**
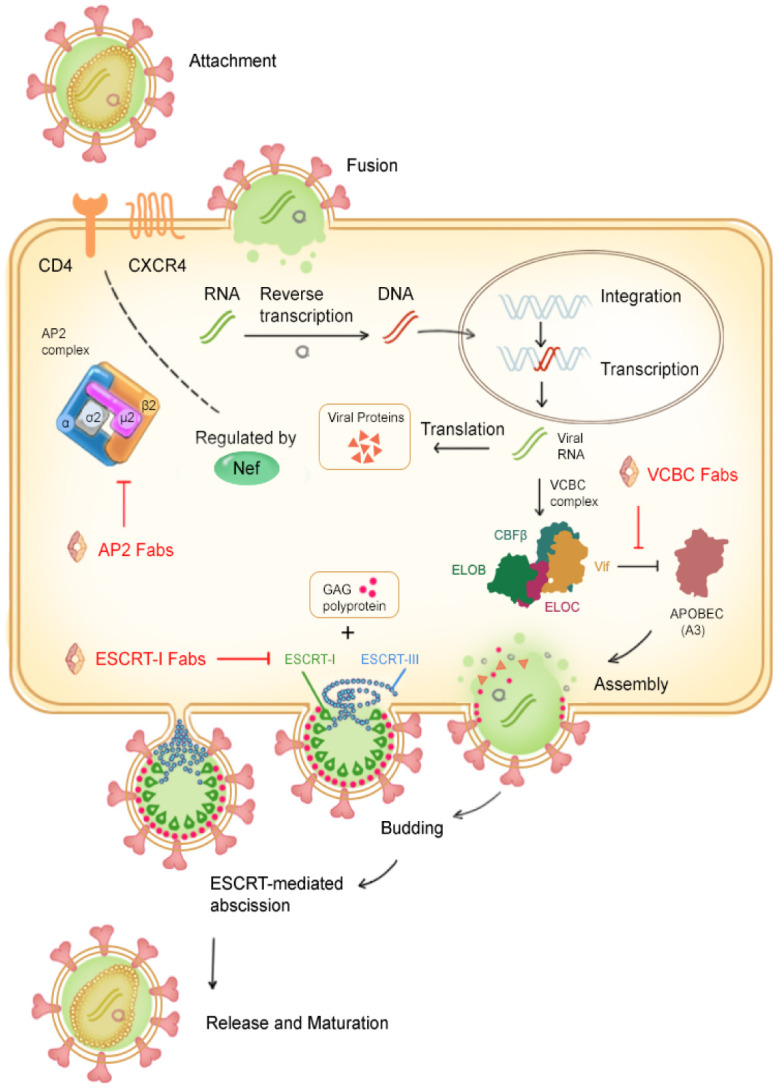
Three protein complexes essential for HIV replication targeted with recombinant antibodies. The antibodies described here are shown in red.

In the case of HIV, three critical dynamic protein assemblies are VCBC [10]; the ESCRT-I [11]; and the clathrin adaptor protein complex 2 (AP2) [12] (summarized in Fig 1). The accessory viral protein Vif (Viral infectivity factor) induces the ubiquitination and degradation of the APOBEC3 (Apolipoprotein B Editing Complex) family restriction factors by binding to the ubiquitin ligase adaptors Elongin B and C (ELOB/C), core binding factor beta (CBF-β) stabilizes the formation of this complex. The HIV structural protein Gag recruits the cellular endosomal sorting complexes required for transport (ESCRT) machinery to facilitate viral budding. The viral accessory protein Nef (negative factor) induces the degradation of the CD4 receptor in the lysosome by binding to the cytosolic tail of CD4 and AP2. To further study these complexes, we have identified Fabs from a naïve human antibody library [13] that recognize conformational epitopes on the complexes with high specificity and high affinity to their cognate antigen.

Here we report well-characterized antibody fragments that recognize HIV-host protein complexes that can aid in obtaining high resolution molecular structures and functional characterization of these complexes. To identify these recombinant antibody reagents, we developed a screening pipeline using quantitative ELISAs and negative stain EM (nsEM) to rapidly and cost effectively identify and test binders, prior to large scale expression and purification of the complexes. The 16 antibody reagents described here are useful “perturbants” of viral function and are available to the scientific community for research purposes.

## Results

### Fab selection

We previously constructed a fully human naïve B-cell Fab phage-display library with a diversity of 4 × 10^10^. This library has been used to successfully generate Fabs against a wide range of protein targets including protease receptors [13], membrane proteins [4, 14] and protein complexes [15].

**Fig 2:**
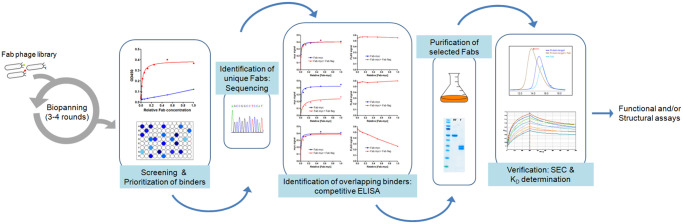
Experimental workflow for antibody selection.

To perform this large discovery effort, we have established a general pipeline for the selection of Fabs using our naïve Fab library. Briefly, three to four rounds of panning against biotinylated antigens (VCBC, ESCRT-I and AP2) are performed according to previously described protocols [13]. After panning, a first screening is conducted by one-point ELISAs with the supernatants of independent selected clones using unpurified Fab from the final round of panning. To prioritize the positive clones and discard false positives, a second ELISA analysis is performed, in this case using serial dilutions of the supernatants (qualitative ELISA), and only the clones with saturating signals are selected for further analysis. The selected clones are then sequenced to identify unique binding Fabs. When needed, an initial characterization of the binding epitope can be done by competitive ELISAs using unpurified FLAG^®^ and myc-tagged Fabs. At this point, only the selected Fabs are expressed and purified from the periplasm of *E*. *coli* BL21 cells. Pure Fabs are used for validation of good binders, the formation of the Fab-antigen complexes is confirmed by elution in one single peak in size exclusion chromatography (SEC). Finally, the affinity of the Fab is determined by biolayer interferometry (BLI) using an Octet instrument (Fig 2). All the Fabs identified in this study have high-affinity binding with equilibrium dissociation constants (K_D_) in the low nanomolar range. The sequences of the complementarity determining regions (CDRs) of the all the Fabs are shown Table 1 and a summary of the selection outcome in S1 Table.

**Table 1:** Fab fragments identified. The sequence of the six complementarity determining regions (CDRs) and the dissociation constants of the Fabs identified in this study. N.D. indicates not determined.

Fab	Epitope group or name	CDRL1	CDRL2	CDRL3	CDRH1	CDRH2	CDRH3	KD (nM)
VCBC complex								
1A3	1	RSSQSLLHSNGYNYLD	GSNRAS	MQGTHWPPA	GGTFSSYAIS	GGIIPIFGTAN	EATYSSGWPYGEYDY	137 ± 29
1B3	1	RSSQRLLHSNGYNYVD	GSNRAS	MAALQTPPT	GGTFSSYAIS	GGIIPIFGTAN	EDGSSGWPYDAFDI	185 ± 22.5
1B10	1	RSSKSLLHSNGYNYLD	GSDRAS	MQALQIPPA	GGTFSSYAIS	GGIIPIFGTAN	TVSSSGWPYDAFDI	193 ± 54.5
3C9	1	TLPSGTFVSAYRIY	SDSDKHRGS	LIWHSTASGYV	GFTFSNYAMS	SSISESGGSTY	AGRYYDFWSGYSPFDY	71 ± 4.2
1D1	Group 2 (Vif complex)	SGSSSNIGSNYVY	NNQRPA	AAWDDSLSGM	GGSISSGGYYWS	GYIYYSGSTN	QGVYYYYGMDV	28 ± 1.7
3F12	Group 3 (Eloc B)	RASQSVSSYLA	GASSRAT	QQYGSSPIT	GSISSSSYYWG	GSIYYSGSTY	SGRGT	419 ± 135
ESCRT-I complex								
CB4	N.D.	TGSSGSIASNYVQ	ATSTRAT	QQYGTSPIT	GGSISSGGYYWG	GSISYSGSTY	TYNLWFGQDAFDI	31 ± 4.9
DB8	N.D.	TGSSGSIASNYVQ	EDNQRPS	QSYDSSSVV	GFTFSSYGMH	AAISYDGSDKY	GDYIWGSYRWGFDY	240 ± 42
DC3	N.D.	RASQSVSSSSLA	AASSRAT	QHYGGSPLLT	GGSINSGLYYWT	GFIFHTGNTY	VESGSFLS	210 ± 17
DD11	N.D.	SGSSSNIGNNYVS	DNNKRPS	GTWDSSLSVVV	GGPISNYYWS	GEINHSGNSN	TKIWLRKEASFQH	110 ± 13
DH5	N.D.	RASQSVGSYLA	DATNRAT	QHRRT	GYTFTDYTIS	GWISSSNGHTK	DGFHSSSWSTYYFDY	300 ± 53
AP2								
CE1	AP2 core	RASQSISSAYLA	GASTRAT	QHRRT	GYTFTDYTIS	GRIIPILGIAN	APTLLGYFDY	11 ± 4.2
CE9	AP2 core	RASQSISSYLN	AASSLQS	QQLNSYPIT	GGTFSSYAIS	GRIIPILGIAN	GGTLLWFGEFDY	12 ± 2.3
BG12	AP2 μ CTD	SGDALPRKYA	EDSKRPS	YSTDSDGDHKGV	GYDFTSFYFQ	IINPLGGSTT	GGSSSSDWALDY	90 ± 6.6
CG7	AP2 μ CTD	SGNKLEDKYVG	DNKRPS	QAWDSGTV	GYTFTSYYIH	IINPSGGST	YGDYADY	34 ± 2.3

### Selection of Fabs against VCBC

The APOBEC3 proteins are cytidine deamidase restriction factors that block the replication of HIV-1 by mutating the viral genome. The HIV accessory protein Vif promotes degradation of APOBEC3 by targeting them for proteolysis by the ubiquitin-proteasome pathway. Vif hijacks the Cullin5-E3 ubiquitin ligase complex (CRL5), composed of Cullin5, RING-box protein 2 (RBX2), and the adaptors ELOB and ELOC. Vif binding requires another host protein, the core-binding factor subunit beta (CBF-β), which stabilizes Vif to form the CRL5-Vif- CBF-β complex [10]. A goal of these studies was to understand how Vif interacts with CRL5 and CBF-β to promote the ubiquitination of APOBEC3 and to possibly disrupt the complex to restore APOBEC3 function. Therefore, we generated Fabs that recognized the native state of the complex.

**Fig 3:**
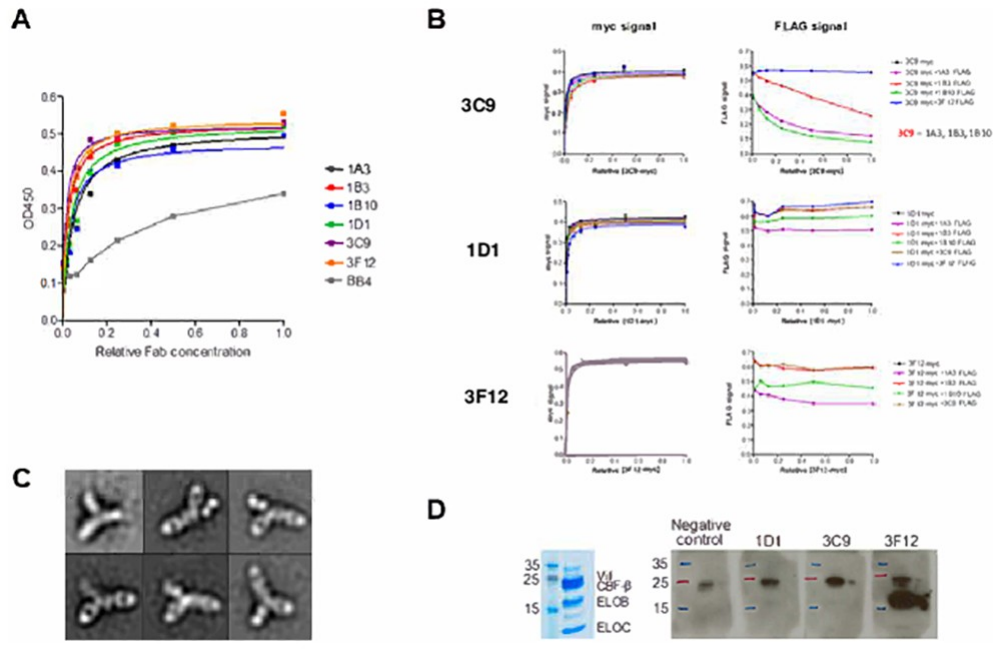
Selection of Fabs against VCBC. ** A**. Qualitative ELISA: Serial dilutions of supernatant of independent clones were added to wells coated with biotinylated VCBC complex. The non-diluted supernatant was considered a relative Fab concentration of 1. The Fab bound to the wells was detected with anti-myc antibody conjugated to horse radish peroxidase. All the Fabs except BB4 displayed saturating signals. **B**. Competitive ELISA: The binding of serial dilutions of 3C9-myc, 1D1-myc and 3F12-myc Fabs supernatants were measured in the presence of saturating constant concentrations of Fab-FLAG. The Fab bound to the wells was detected with anti-myc (left panel) or anti-FLAG (right panel) antibodies. **C**. Representative negative stain 2D class averages of VCBC in complex with the three selected Fabs (3C9, 1D1 and 3F12). **D**. Detection of VCBC by western blot using 3C9-myc, 1D1-myc and 3F12-myc as primary antibodies and anti-myc-HRP as secondary antibody. Only 3F12 binds to the denatured form of VCBC and recognizes a linear epitope in ELOB. One blot without primary antibody was used as negative control.

The biotinylated VCBC complex (Vif- CBF-β-ELOB-ELOC) in its native, soluble state was used for biopanning and Fab selection. After four rounds of biopanning, 380 independent clones were screened by single point ELISA with, forty showing a signal ten times over background in the ELISA. The heavy and light chain expression cassettes of all 40 VCBC binding clones were DNA sequenced and seven had unique sequences. Phage infected E. coli TG1 cells were grown in selection media and Fab expression was induced and supernatants were used to carry out quantitative ELISA assays. Six of the Fabs (1A3, 1B3, 1B10, 1D1, 3C9 and 3F12) showed dose dependent, saturating quantitative ELISA curves. One Fab, BB4, displayed a non-saturating quantitative ELISA curve and was confirmed to be a weak binder (Fig 3A). BB4 binding kinetics were not consistent with specific binding therefore, we did not pursue BB4 further. The six most promising Fabs were subsequently shown to have binding affinities in the low nanomolar range determined by BLI. The sequences of the CDRs of the Fabs are shown Table 1 and a summary of the selection outcome in S1 Table.

We classified the Fabs in three different epitope groups based on the results from a competitive ELISA format (Fig 3B). For this experiment, each Fab was expressed with two different tags, myc or FLAG and supernatants from *E*. *coli* TG1 cells expressing the Fabs was sufficient to carry out the ELISAs. Biotinylated VCBC was immobilized in wells and ELISAs were performed using 3C9-myc at varying concentrations and one of the other Fab-FLAG (1A3, 1B3, 1B10 or 3F12) at saturating constant concentration. 3C9-myc binding is not affected by the presence of any Fab-FLAG (Fig 3B and B3C9. left panel). 1A3-FLAG, 1B3-FLAG and 1B10-FLAG binding decreases when the concentration of 3C9-myc increases. 3F12-FLAG binding was constant at all 3C9-myc concentrations (Fig 3B, right panel). Similar experiments were performed with 1D1-myc (Fig 3B, B1D1) or 3F12-myc (Fig 3B and B3F12) at varying concentrations and one of the other Fab-FLAG at saturating constant concentrations. These results suggest that 1A3, 1B3, 1B10 and 3C9 have overlapping epitopes (class 1) and 3C9 has a higher affinity for VCBC that the other competing Fabs. 1D1 and 3F12 have unique epitopes (class 2 and 3 respectively).

Based on these studies 3C9, 1D1 and 3F12 were selected for large scale protein production and further analysis. These three antibodies form stable complexes with VCBC as confirmed by nsEM (Fig 3C). The six representative 2D class averages of VCBC in complex with 3C9, 1D1 and 3F12 show that the three Fabs recognize independent sites on the VCBC complex in agreement with the competitive ELISA assays.

To determine if the Fabs recognize the native structure of the VCBC complex, the Fabs were used as primary antibodies to detect VCBC by Western blot. Fabs 3C9 and 1D1 do not bind to denatured VCBC suggesting that they should bind a three-dimensional epitope in the complex. In contrast, Fab 3F12 recognizes a linear epitope in ELOB (Fig 3D).

**Fig 4:**
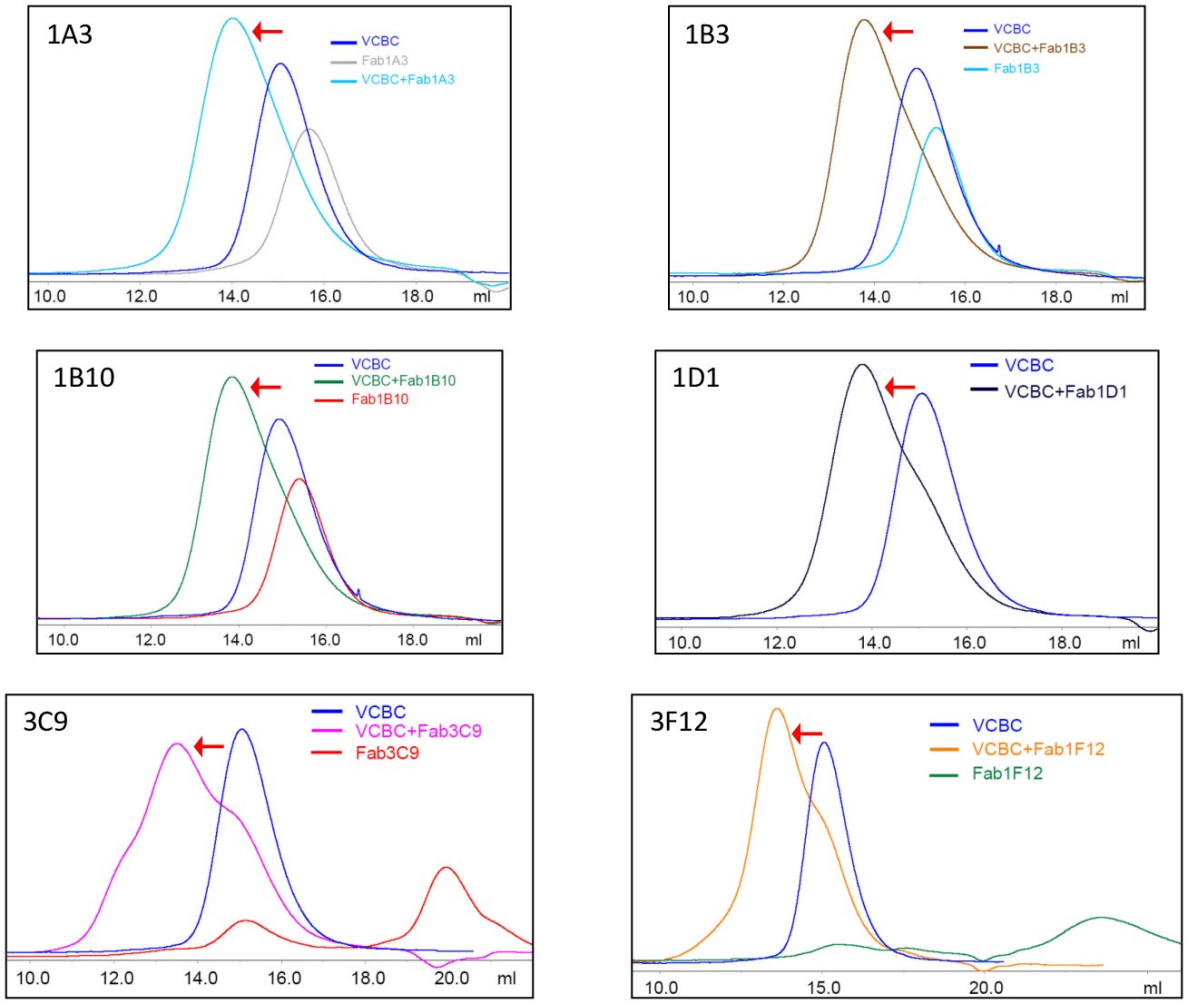
Elution profiles of VCBC complexed with six different Fabs. The mixtures of VCBC and Fab at 1:1 stoichiometry were loaded onto Superdex-200 analytical columns and UV profiles of the eluates were compared to those of VCBC and individual Fabs. X-axis indicates volume (mL) eluted, Y-axis indicated absorbance at 280nm. The shift in elution volume indicates that VCBC forms a stable complex with the Fabs at 1:1 ratio. The elution profile of Fab 1D1 alone is shown in S1 Fig.

Fabs 1A3, 1B3, 1B10, 1D1, 3C9 and 3F12 were then tested to see if they could form stable complexes under gel filtration conditions. Stoichiometric amounts of each Fab was mixed with VCBC and subjected to size exclusion chromatography. The chromatogram of each mixture was then compared to the chromatograms of the Fab alone and VCBC alone (Fig 4, S1 Fig). All six Fabs show well separated, stable complex peaks with VCBC. While 1A3, 1B3 and 1B10 display symmetrical peaks for the complex, 1D1, 3C9 and 3F12 exhibit asymmetry in the Fab:VCBC complex suggesting an equilibrium between the Fab and the complex resulting in separate shoulders from the main peak. These three Fabs also show non-specific binding to the chromatography resin as evidenced by two elution peaks and large elution volumes (Fig 4, S1 Fig).

Fabs 3C9, 1D1 and 3F12 have been used for functional and structural characterization of A3-Vif interactions. Intriguingly, 3C9 and 1D1 were shown to inhibit Vif-mediated APOBEC3 ubiquitination and also facilitated determination of a low-resolution EM structure of the VCBC complex [15].

Membrane budding is an essential part of the HIV life cycle. HIV recruits the host ESCRT machinery to bud from the plasma membrane. HIV-1 p6 Gag contains two motifs that bind and recruit early-acting ESCRT factors. The PTAP domain binds the TSG101 subunit of the heterotetrameric ESCRT-I complex and the “YPXL” (Tyr-Pro-X-Leu) that binds the ESCRT factor ALIX [16]. These interactions result in the recruitment of ESCRT-III and VPS4 complexes which together constrict membranes and mediate fission [17].

### Selection of Fabs against ESCRT-I

Specific Fabs against HIV-1 p6 Gag, the ESCRT factors, or the complex can aid the design of new inhibitors of HIV budding and help characterize existing inhibitors. We wanted to determine if the antibodies that bound ESCRT-I would reduce viral budding. For these purposes, we used biotinylated ESCRT-I complex in its native, soluble state for Fab selection. ESCRT-I contains a single copy of the TSG101 (also referred to as VPS23) and VPS28 subunits and single copies of one of the different isoforms of VPS37 and MVB12.

**Fig 5:**
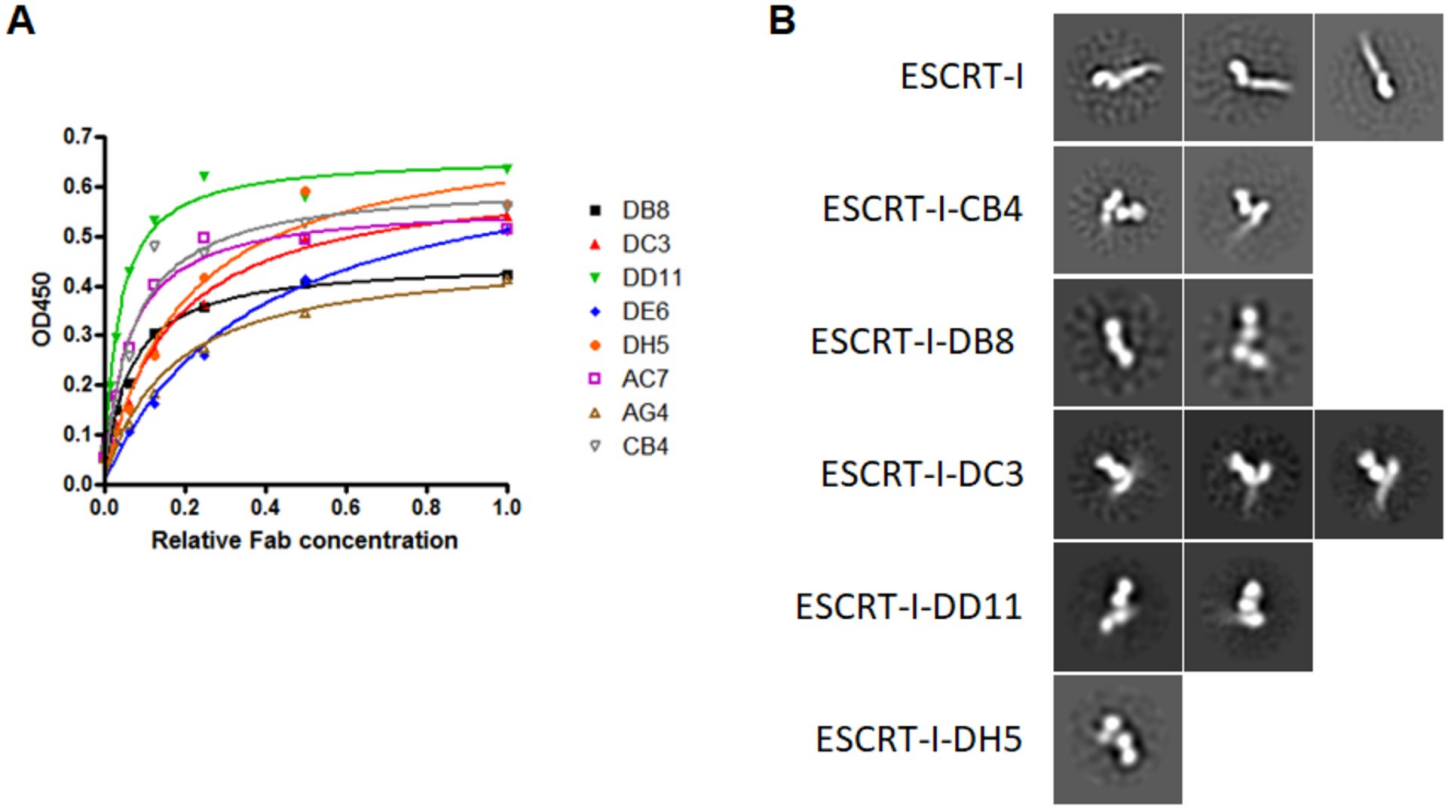
Selection of Fabs against ESCRT-I. **A**. Representative qualitative ELISA of the 8 selected Fabs. Serial dilutions of supernatant of independent clones were added to wells coated with biotinylated ESCRT-I complex. The non-diluted supernatant was considered a relative Fab concentration of 1. The Fab bound to the wells was detected with anti-myc antibody conjugated to peroxidase. **B**. Representative negative stain 2D class averages of ESCRT-I complex with and without specific Fabs.

After three rounds of panning, 380 independent clones were screened by single point ELISA with 45 of these showing a signal ten times over background in the ELISA. The heavy and light chain expression cassettes of all 45 ESCRT-I binding clones were DNA sequenced and 24 had unique sequences. Phage infected *E*. *coli* TG1 cells were grown in selection media and Fab expression was induced and supernatants were used to carry out quantitative ELISA assays. Fab binding was validated by qualitative ELISA and 16 Fabs were confirmed as binders. The eight Fabs with highest signal were then characterized using the quantitative ELISA format with each Fab displaying a dose dependent, saturating quantitative ELISA curve (Fig 5A). Five (CB4, DB8, DC3, DD11, DH5) of the eight Fabs eluted in a single peak with ESCRT-I by size exclusion chromatography, presumably due to the binding affinities of DE6, AC7 and AG4 being insufficient to form stable complexes under the column chromatography conditions (S2 Fig). The binding affinities of the Fabs ranged from 30 to 300 nM and their K_D_ values and CDR sequences are shown Table 1. A summary of the selection outcome in S1 Table. The five selected Fabs were considered excellent candidates for structural studies of ESCRT-I interactions and the stability and rigidity of the complexes with ESCRT-I was confirmed by nsEM (Fig 5B).

**Fig 6:**
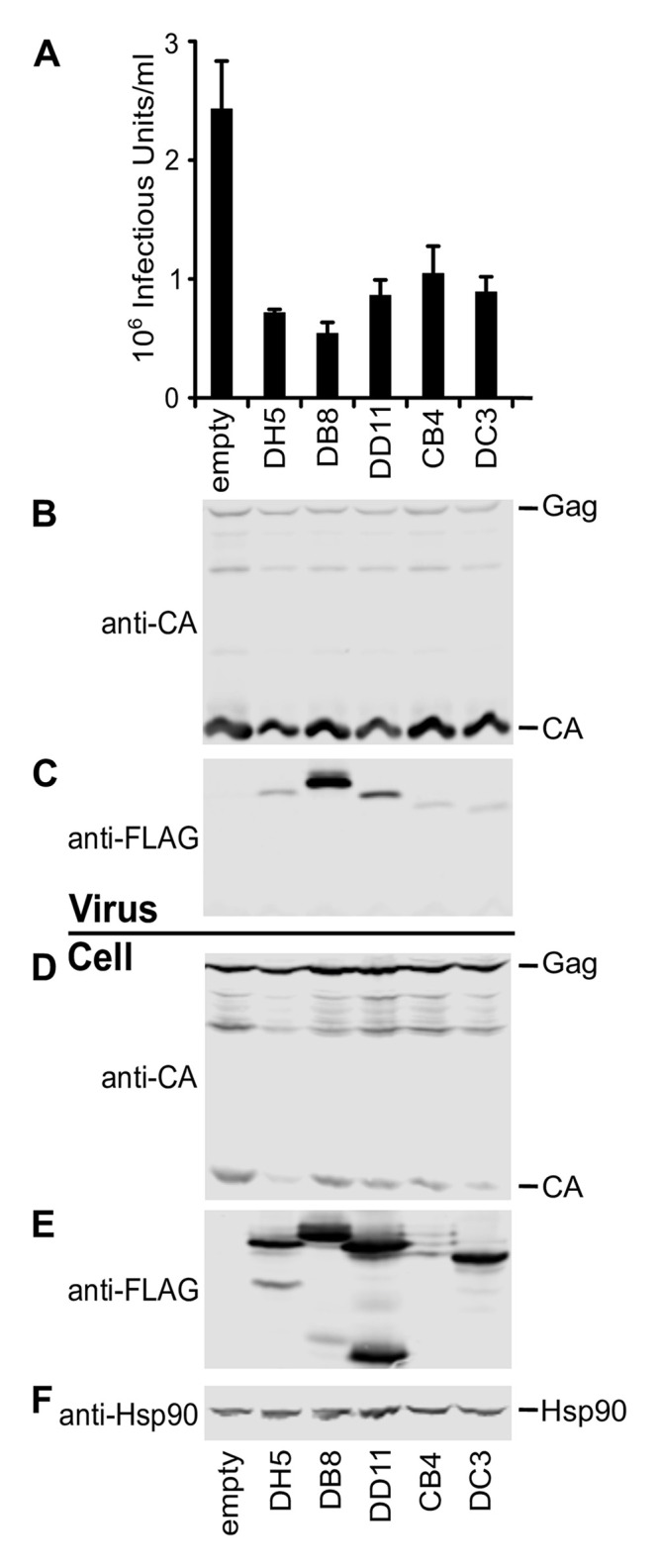
Effects of ESCRT-I binding protein expression on HIV-1 release and infectivity. Virus-producing cells were co-transfected with empty plasmid (labeled ‘Empty’), or antibodies DH5, DB8, DD11, CB4, and DC3 as described in material and methods. **A**. Infectious units released from transfected cells 293T cells, measured by titration on MT4 cells as described in material and methods. Error bars show the standard deviation of 3 independent experiments. **B-F**. Western blots. **B**. Western blot showing levels of virion-associated HIV Gag (Gag) and capsid (CA) proteins (**B**, “Virus”), and antibodies incorporated into virions (**C**, “Virus”). Designated intracellular viral and cellular proteins in cells expressing HIV-1 (**D** “Cell”) and antibodies (**E**, “Cell”). Positions of HIV-1 Gag polyprotein precursor and the mature HIV-1 CA protein are indicated on the right of the membrane, lanes are labeled with antibodies tested. **F**. Hsp90, which was used as loading control.

We next asked whether expression of any of the Fabs (CB4, DB8, DC3, DD11, DH5) in the appropriate format could affect ESCRT-I mediated budding of HIV-1 in cells. Single-chain variable fragment (scFv) constructs of each Fabs was generated for intra-cellular expression in mammalian cells. HEK293T cells were co-transfected with either an empty plasmid (control) or FLAG-tagged versions of the scFvs, and an HIV-1_NLENG-IRES_ proviral expression construct expressing infectious HIV-1 with a GFP reporter gene [18]. Expression of scFvs that bind ESCRT-I reduced infectious units released from cells between ~2.3-fold (CB4, Fig 6A and 6B) and ~4.5-fold (CB8, Fig 6A and 6B) compared to the control (Fig 6A and 6B, lane labeled “empty”). FLAG-tagged scFv were packaged into the virions, as shown in Fig 6C. Expression of DH5 and DC3 scFvs also reduced cellular Gag and capsid (CA) levels relative to the control with no scFv (Fig 6D) indicating some cytotoxicity. FLAG-tagged scFv remained in the cells, showing some degradation Fig 6E. Hsp90 was used as a loading control and levels of Hsp90 were similar in all samples (Fig 6F). All the scFv tested were found in virus preparations, suggesting that they are incorporated into budding virions.

### Selection of Fabs against AP2 complex

Nef is an HIV accessory protein essential for HIV-1 infectivity that has multiple functions. One of the best characterized functions is the downregulation of CD4, the cellular receptor for the virus, from the surface of infected cells. Nef interacts with Adaptor Protein complexes (AP). APs are a family of hetero-tetramers that recognize cytoplasmic sorting motifs in transmembrane proteins and package them into clathrin-coated vesicles for intracellular transport [12]. Nef downregulates CD4 by formation of a tripartite complex with the clathrin-associated adaptor protein complex AP2 at the plasma membrane, leading to accelerated CD4 endocytosis and lysosomal degradation [19]. Structural studies of CD4-Nef-AP2 indicate that Nef hijacks clathrin- and AP2-dependent endocytosis, which removes CD4 from the cell surface [20]. The goal of these studies was to generate conformationally selective Fabs that recognize the complex or one of its components to aid structural studies and to identify epitopes on Nef that would lead to its inhibition.

AP2 is composed of α, β2, μ2 and σ2 subunits. We performed Fab selections against the biotinylated native AP2 core comprised of the N-terminal ‘trunk’ domains of α and β2 together with the whole μ2 and σ2 subunits (~200 kDa), and against the biotinylated native C-terminal domain of the medium μ subunit (AP2 muCTD, ~ 32kD) that is responsible of binding YXXΦ signals in endogenous cargoes.

**Fig 7:**
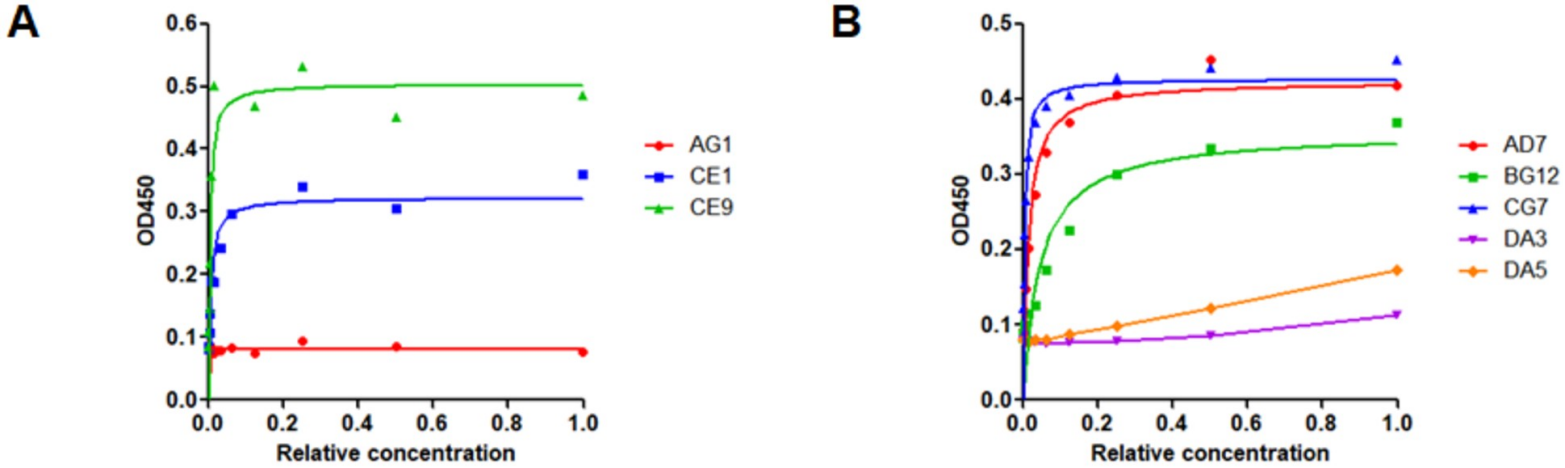
Selection of Fabs that bind the AP2 complex. For the quantitative ELISAs serial dilutions of supernatant from independent clones were added to wells coated with biotinylated AP2 core **A**. and AP2 muCTD **B**. The non-diluted supernatant was defined as having a relative Fab concentration of 1. The Fab bound to the wells was detected with anti-myc antibody conjugated to peroxidase. Fabs CE1 and CE9 for AP2 core and Fabs AD7, BG12 and CG7 for AP2 muCTD displayed dose-dependent saturating signals.

After four rounds of panning, 380 independent clones of each target were screened by single point ELISA. Nine clones had ELISA signals greater than ten-fold over background for AP2 core and six clones for AP2 muCTD. DNA sequencing of the heavy and light chain expression cassettes of each of the binding clones yielded three AP2 core clones and five AP2 muCTD clones with unique sequences. Using the quantitative ELISA format, clones CE1 and CE9 showed dose dependent, saturating quantitative ELISA curves for AP2 core while BG12 and CG7 showed dose dependent, saturating quantitative ELISA curves for AP2 muCTD (Fig 7A). AD7 did not express well and was not pursued further. The four most promising Fabs (CE1, CE9, BG12 and CG7 were then produced in reagent quantities and shown to have binding affinities in the low nanomolar range determined by BLI. Fabs BG12 and CG7 also form stable complexes with the AP2muCTD (Fig 7B). The sequences of the CDRs of the Fabs are shown Table 1 and a summary of the selection outcome in S1 Table.

**Fig 8:**
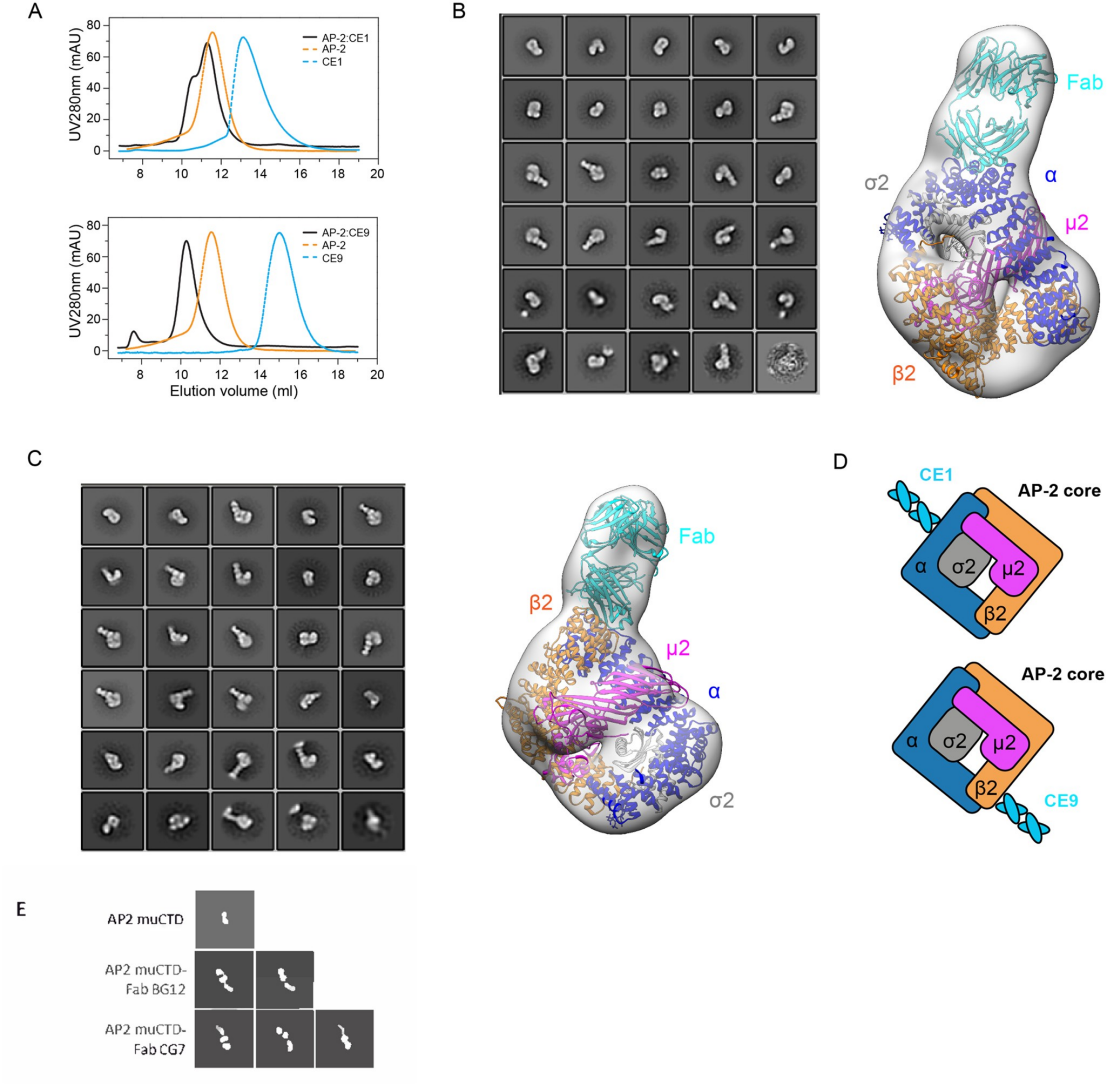
Characterization of Fabs that bind the AP2 complex. **A**. SEC profiles of AP2 core:CE1 Fab (black in upper panel), AP2 core:CE9 Fab (black in lower panel), AP-2 core alone (dashed orange) and Fab alone (dashed cyan) on a Superdex 200 10/300GL column. Single particle negative stain EM study of AP-2 core: CE1 Fab. **B**. or AP-2 core:CE9 Fab **C**. The left panel is 2D class averages for AP-2:Fab complex. The right panel showed that the atomic structures of a Fab (PDB: 3SQO and chain H,L in cyan) and an AP-2 core (PDB: 2VGL) were docked in the density map as rigid bodies (AP2 α in blue, β2 in orange, μ2 in magenta and σ2 in gray). **D**. Cartoon representation of Fabs (CE1 in upper panel, CE2 in lower panel) binding to AP-2 core. **E**. Representative negative stain 2D class averages of AP2 muCTD with BG12, CG7 or alone.

To determine if the four tight binding Fabs form stable complexes that could be visualized using EM complexes of CE1 and CE9 were made with AP2 under negative stain conditions. As seen in the negative stain class averages, CE1 and CE9 serve as fiducial markers with and form stable complexes with for the AP2 as shown in Fig 8.

## Discussion

The structural study of host-HIV interactions has been challenging due to the high degree of conformational flexibility of these proteins. Highly specific and potent recombinant antibody fragments that bind to distinct conformational states of HIV-host complexes are good reagents for structural and functional characterization of these interactions and can lead to new strategies for therapeutic intervention. As we and others have shown, antibody fragments can facilitate structural biology studies as specific binding proteins to accelerate crystallization; to facilitate single particle cryo-EM studies by acting as fiducial markers and overcoming preferred orientations; and to reduce the flexibility of dynamic proteins and increase the structural homogeneity for biophysical studies [4, 14, 21].

Antibodies are powerful research tools used in multiple research applications. In the last two decades, phage display has emerged as a powerful methodology for the *in vitro* generation of recombinant antibodies. The main advantages of the *in vitro* selections over the traditional hybridoma methods are the complete elimination of animals from the production process and the control of the conditions of selection, that allows the identification of antibodies that bind specific protein conformations including native antigens. Furthermore, recombinant antibodies are fully renewable. The sequence encoding the antibody is immediately available and it provides advantages for engineering the selected antibody by altering the format (e.g., full-length antibody, Fab or scFv) increasing the affinity by affinity maturation and introducing additional protein tags for purification or identification. Finally, the generation of recombinant antibodies can be adapted to robotics for high-throughput production.

To generate antibodies to protein complexes, we used a human, naïve Fab-displayed library to provide a rich source of antibody diversity. Using this library, we have successfully generated recombinant Fabs that are useful for both functional and structural analysis against a wide range of protein targets including proteases [22], membrane proteins [13, 14] and protein complexes.

Here, we describe use of our Fab library to generate a panel of recombinant Fabs against HIV-related targets: VCBC; ESCRT-I and the clathrin adaptor complex AP-2. All of the Fabs form stable and rigid complexes and have aided structure determination of the protein targets by nsEM.

In addition to the structural applications, these antibodies can aid functional characterization of target protein complexes. For the cellular studies we expressed the scFv version of the Fabs intracellularly, with VH and VL fragments joined by a flexible linker peptide. This approach was used for the VCBC antibodies, with 1D1 and 3C9 scFvs being used to probe and inhibit the function of Vif [15]. The results obtained with VCBC Fabs and scFv demonstrate the potential value of these antibodies as tools, or “perturbants” for studying HIV-host interactions. Expression of the scFvs in human cells that bind ESCRT-I and reduced HIV-1 infectious units released from cells ~2-5-fold. HIV-1 release is highly dependent on the functioning of ESCRT-I. Knock out of the ESCRT-I component TSG-101 was found to reduce viral titers by ~80-fold [23]. Our finding that ESCRT-I binding scFvs modestly reduced viral titers indicates that these scFvs do not completely block ECSRT-I function. One possible explanation is that the scFvs bind in regions that are not essential for ESCRT-I Gag interaction and downstream recruitment of late acting ESCRT-III proteins. Nevertheless, all the ESCRT-I binding scFvs were incorporated into virus particles [24] (Fig 6C and 6E).

The technology described here can be applied to other viral-host targets. Successful selection of antibodies is dependent on highly pure and homogeneous antigen. The antibodies presented in this paper were generated at low throughput, but this approach can be adapted to robotics for high-throughput production.

In conclusion, we have established a rapid protocol for identifying phage displayed antibodies and present a panel of renewable, specific, and well-characterized reagents generated using this protocol. These antibodies can be used to study host-virus interactions that will help to provide new probes for the investigation of HIV.

## Materials and methods

### Antigen preparation

Successful selection of recombinant antibodies requires high-quality and stable antigens. Antigens used in this study were recombinantly produced in *E*. *coli*. VCBC [25], ESCRT-I [11], and AP2 [26] were prepared as described. To obtain a homogenous VCBC, His-thioredoxin-tagged CBFβ was co-expressed with Vif and ELOBC in *E*. *coli* strain BL21-star (DE3) and purified by immobilized metal affinity, heparin and size exclusion chromatography under the buffer conditions containing 20 mM HEPES pH 7.5, 0.5 M NaCl and 10% glycerol. During the VCBC purification, His-thioredoxin tag was removed from VCBC by treating TEV protease and Superdex-75 prep column was used for size exclusion chromatography (SEC). For phage display selections, the protein target needs to have a tag to enable immobilization on a surface, all the purified antigens were biotinylated using EZ-Link NHS-Chromogenic-Biotin (Pierce). The ratio of biotinylation was spectrophotometrically quantified following the manufacturer’s instructions. In general, antigens with one ratio of 1:1 or 2:1 (biotin: protein/complex) were used for selections. The efficiency of binding to streptavidin magnetic beads was tested before the panning.

### Phage display panning

The Fab selections were done as described previously [13]. A human naïve B cell Fab phage-displayed library [13] was used to identify specific Fabs against our targets. The buffer used during panning was specific for antigen. Panning was accomplished in three or four rounds using streptavidin magnetic beads (Invitrogen) coated with the biotinylated antigen. The stringency of panning was increased in each round by decreasing protein concentration and increasing the number of washes. In second, third and fourth rounds negative selections against streptavidin beads were done to eliminate streptavidin binding phage. The panning was monitored by titration of bound phages following each round. In the final round of selection, Fabs were produced from individual clones in a 96-well format and were screened for binding to the antigen by ELISA, described below. Clones with a positive signal in ELISA were analyzed by BstNI restriction analysis and DNA sequencing to identify the unique clones. A summary of the selection outcome in S1 Table.

### ELISA

The design of our naïve Fab library allows us to accelerate the screening process by using unpurified Fabs in culture supernatant for ELISA. The phagemid contains one amber codon between the heavy chain of the Fab and phage gene 3 (g3p) that allows both the display of the Fab when it is produced as a fusion protein Fab-g3p, and the production of soluble Fab in an amber suppressor strain such as TG1. A low percentage of these soluble Fabs leak into media during phage propagation. The leaked Fabs are relatively pure and can be used directly in ELISA analyses.

The ELISAs were done using standard procedures. Briefly, MaxiSorp^®^ (Nunc) plates were coated with 50 μL of 5 μg/mL of streptavidin (Promega) in PBS overnight at 4°C. Plate was washed with PBS and blocked with 2% BSA-PBS. Plate was washed with protein buffer and 50 μL of 10–15 μg/mL of biotinylated antigen (in protein buffer with 1% BSA) was added to the wells and incubated for 45 min. The plate was washed with protein buffer and 50 μL of Fab supernatants in 1% BSA were added to each well and incubated for 1 hour. Wells were washed with protein buffer-0.05%Tween and the Fab was detected with anti-myc antibody conjugated to peroxidase (Roche) and TMB reagent (Pierce). The absorbance was determined at 450 nm using a microplate reader.

For qualitative ELISAs the protocol was the same as above except that serial dilutions of the Fab supernatants were used instead of a single concentration. The Fabs expressed from our phage display library have a myc tag in the C-terminus of the heavy chain that is used for the detection of the Fab in the ELISAs. For competitive ELISAs, the myc tag in the phagemid was replaced with the Flag tag for independent detection of the two competing Fabs. Equal volumes of Fab-myc and Fab-FLAG solutions were added for each binding reaction. Fab-myc and Fab-FLAG were detected with anti-myc antibody conjugated to peroxidase (Roche) and anti-FLAG M2-peroxidase (Sigma) respectively.

### Fab expression and purification

The Fabs expressed from our library have one hexahistidine tag in the C-terminus of the heavy chain that allows the affinity purification with nickel resins.

Fabs were expressed and purified in *Escherichia coli* BL21 (DE3) Gold (Stratagene). Cultures were grown in 1 L of 2x YT containing 100 μg/mL ampicillin and 0.1% glucose at 37°C and 200 rpm to an optical density at 600nm of 0.6. The protein expression was induced with the addition of 1mM IPTG and grow overnight at 20°C. The cells were harvested by centrifugation and the cell pellet was resuspended in ice-cold TES (0.2 M Tris pH 8, 0.5 mM EDTA, 0.5 M sucrose). The cell suspension was mixed with the same volume of ice-cold distilled H_2_O and incubated on ice for 30 min. The solution was then pelleted and the supernatant (periplasmic fraction) was used for the purification.

The periplasmic fraction was applied to a column containing 1mL of Ni-NTA beads prewashed with wash buffer 1 (50 mM Tris pH 8, 250 mM NaCl). The column was washed with 20 column volumes of wash buffer 2 (50 mM Tris pH 8, 500 mM NaCl, 20 mM Imidazole) and the Fab was eluted with 3 column volume of elution buffer (50 mM Tris pH 8, 500 mM NaCl, 500 mM Imidazole). The purified Fab was dialyzed overnight at 4°C against PBS buffer pH 7.4 and analyzed by SDS-PAGE.

### Size exclusion chromatography

VCBC was mixed with individual Fabs in a 1:1 ratio and injected into a Superdex-200 analytical column equilibrated with a buffer containing 20 mM HEPES pH 7.5, 0.5 M NaCl and 10% glycerol. The UV profile of the mixture was superimposed with those of VCBC and Fab to compare the elution volume.

### Determination of K_D_ values

Kinetic constants for each Fab were determined using an Octet RED384 instrument (FortéBio, Fremont, CA). Four concentrations of Fab was tested for binding to the biotinylated antigen immobilized on FortéBio streptavidin SA biosensors. All measurements were performed at room temperature in 384-well microplates and the running buffer was the protein buffer with 0.1% (w/v) bovine serum albumin (BSA) and 0.02% (v/v) Tween 20. The conditions of the experiment (baseline, association, and dissociation times) were adjusted for each antigen-Fab pair. Data were analyzed using a 1:1 interaction model on the FortéBio data analysis software 8.2.

### Negative stain electron microscopy

Purified antigens and Fabs were mixed at a 1:1 molar ratio and incubated on ice for one hour. Immediately prior to grid preparation, samples were diluted to either 100 nM for AP2M160 and ESCRT-I complexes or 50 nM for AP2core complexes. Grids of antigen alone and antigen-Fab complexes were prepared for negative-stain EM following established protocols [27]. Briefly, 2.5 μL of sample was applied to glow-discharged carbon coated Cu EM grids (Ted Pella Inc., Redding, CA) and stained with 0.75% uranyl formate. Negatively stained EM grids were imaged on a T20 microscope (FEI Company) operated at 200 kV with a nominal magnification of 50,000x using a TemF816 8K × 8K CMOS camera (TVIPS GmbH, Gauting, Germany), corresponding to a calibrated pixel size of 1.57 Å on the specimen. All images were binned by 2 for further image processing, resulting in a pixel size of 3.14 Å. Defocus values were determined using gctf and particles were picked using Gautomatch with a Gaussian template [28]. Two-dimensional class averages were generated with RELION 2 [29].

### Western blot

Antigen (1μg) was separated in an SDS-PAGE gel using reducing conditions and transferred to a PVDF membrane. The membrane was blocked and incubated with the Fabs (1μg/mL) for 2 hours. The Fabs were detected using an anti-myc antibody conjugated to peroxidase (Roche).

### Mammalian cell culture

Human embryonic kidney (HEK) 293T cells were obtained from ATCC (Manassas, VA) and cultured in D-MEM (ThermoFisher) containing 10% FBS, penicillin (100 U/ml) and streptomycin (0.1 mg /ml), at 37°C and 5% CO_2_. MT-4 T cells [30] were obtained from the NIH AIDS Reagent Program and cultured in RPMI (ThermoFisher) containing 10% FBS, penicillin (100 U /ml) and streptomycin (0.1 mg/ml), at 37°C and 5% CO_2_. Cells were tested for mycoplasma contamination every 3 months using the MycoAlert Mycoplasma Detection Kit (Lonza).

### ESCRT-I binding scFv expression in mammalian cells and virion production assays

Antibodies that bind ESCRT-I were expressed in 293T cells and effects on ESCRT-driven HIV-1 release were examined over the following time course: The day before transfection, cells were seeded at 3 × 10^5^ cells/well in 6-well plates. Cells were co-transfected with 1 μg HIV-1 GFP reporter plasmid pNLENG-IRES [18] and 2 μg pCMV4 scFv expressing plasmids using the polyethyleneimine (PEI, Polysciences, 3 μl of PEI (1mg/ml)/ μg DNA) method. Medium was changed after 12 h. 40 h post transfection, cells and culture supernatants were harvested for analysis. Released HIV-1 virions were collected from the culture supernatants by centrifugation through a 200 μl 20% sucrose cushion for 90 min at 21,000 × *g*, 4°C, and denatured by adding 50 μl 1 × Laemmli buffer and boiling for 5 min. Cells were lysed on ice in 100 μl cold lysis buffer (50 mM Tris pH 7.4, 150 mM NaCl, 1% Triton X-100, protease inhibitors) and 100 μl 2x Laemmli buffer, and boiled for 10 min. HIV-1 titers were analyzed using MT-4 T cells seeded at 200,000 cells per well in 96 well plates and inoculated with serial dilutions of HIV-1 GFP reporter virus (200 μl medium in total per well). 100 μl of the medium was replaced the 16 h post infection and Dextran sulfate was added in a final concentration of 100 μg/ml to prevent spread of infection. Infected cells (% GFP positive cells) were counted by FACS analysis using a Celesta Flow cytometer (BD Biosciences) 72 h post infection.

## Supporting information

S1 Fig Size exclusion chromatography of fab 1D1 alone on Sepharose 200. Absorbance at 280 nM is shown.
(DOCX)

S2 Fig Size exclusion chromatography of fabs alone and with ESCRT-I (hE1). (DOCX)

S1 Table Summary of Fab selections. (DOCX)

S1 Raw images (DOCX)
