# Supplementary material for: Identification of recombinant Fabs for structural and functional characterization of HIV-host factor complexes
Source: PLoS One. 2021 May 13;16(5):e0250318. doi: 10.1371/journal.pone.0250318 (PMC8118348; doi:10.1371/journal.pone.0250318)
Supplement: S1 Fig — Absorbance at 280 nM is shown. (DOCX) [file pone.0250318.s001.docx]

S1 Fig. Size exclusion chromatography of fab 1D1 alone on Sepharose 200. Absorbance at 280 nM is shown.
