# Supplementary material for: Identification of recombinant Fabs for structural and functional characterization of HIV-host factor complexes
Source: PLoS One. 2021 May 13;16(5):e0250318. doi: 10.1371/journal.pone.0250318 (PMC8118348; doi:10.1371/journal.pone.0250318)
Supplement: S2 Fig — (DOCX) [file pone.0250318.s002.docx]

S2 Fig. Size exclusion chromatography of fabs alone and with ESCRT-I (hE1)

Raw gels

Fig 3 D.


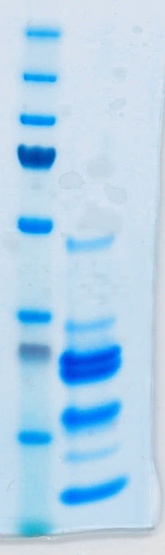


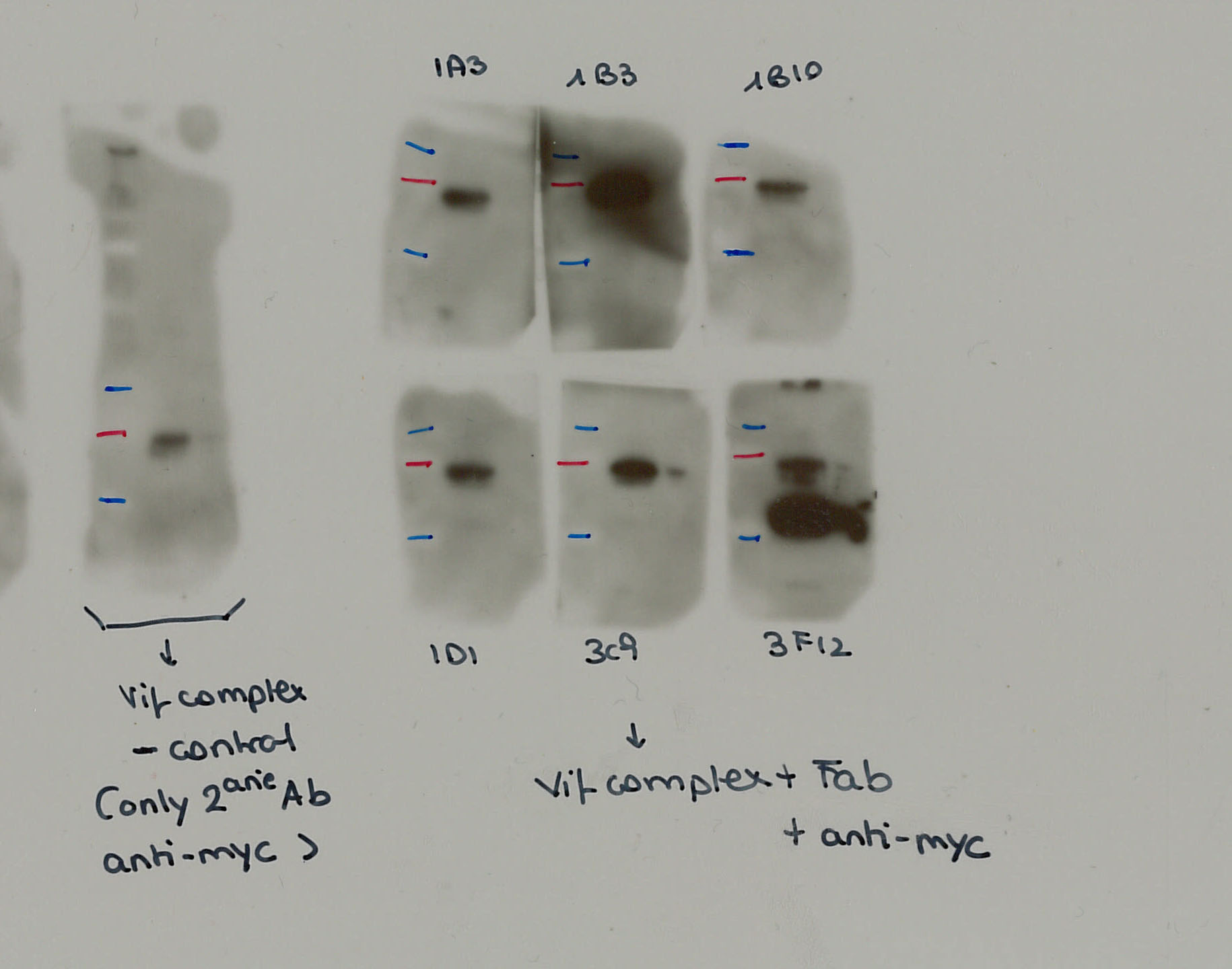


Raw data gels for Fig 6
