# Supplementary material for: Identification of recombinant Fabs for structural and functional characterization of HIV-host factor complexes
Source: PLoS One. 2021 May 13;16(5):e0250318. doi: 10.1371/journal.pone.0250318 (PMC8118348; doi:10.1371/journal.pone.0250318)
Supplement: S1 Table — (DOCX) [file pone.0250318.s003.docx]

S1 Table: Summary of Fab selections

| **Target** | **Rounds of panning** | **Number of clones screened** | **Initial hits** | **Unique clones** | **Tight binders** |
| --- | --- | --- | --- | --- | --- |
| VCBC complex | 4 | 380 | 40 | 7 | 1A3, 1B3, 1B10, 1D1, 3C9, 3F12 |
| ESCRT-1 complex | 3 | 380 | 45 | 24 | CB4, DB8, DC3, DD11, DH5 |
| AP2 core | 4 | 380 | 9 | 3 | CE1, CE9 |
| AP2 muCTD | 4 | 380 | 6 | 5 | BG12, CG7 |
