# Supplementary figures and images for: Identification of recombinant Fabs for structural and functional characterization of HIV-host factor complexes
Source: PLoS One. 2021 May 13;16(5):e0250318. doi: 10.1371/journal.pone.0250318 (PMC8118348; doi:10.1371/journal.pone.0250318)

Raw gels

Fig 3 D.


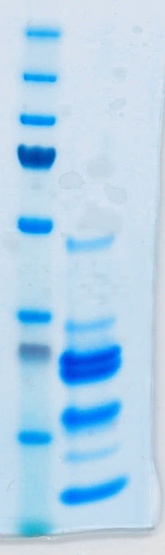


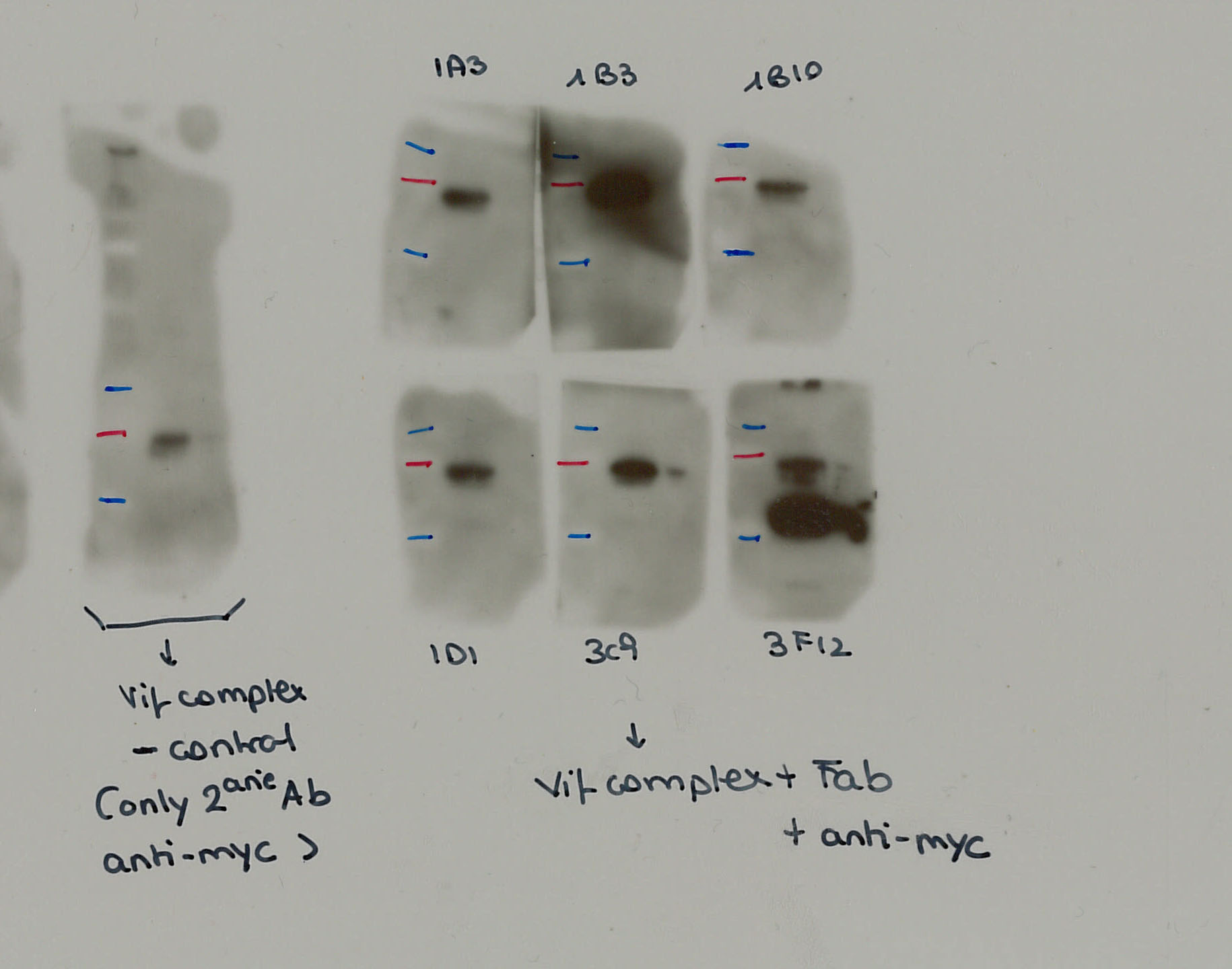


Raw data gels for Fig 6

Supplement: S1 Raw images — (DOCX) [file pone.0250318.s004.docx]
